# Intervening for sustainable change: Tailoring strategies to align with values and principles of communities

**DOI:** 10.3389/frhs.2022.959386

**Published:** 2023-01-18

**Authors:** Allison Metz, Kirsten Kainz, Annette Boaz

**Affiliations:** ^1^ School of Social Work, University of North Carolina at Chapel Hill, Chapel Hill, NC, United States; ^2^ Department of Health Services Research and Policy, London School of Hygiene and Tropical Medicine, London, England

**Keywords:** strategies, values, implementation, tailoring, mental models

## Abstract

This paper presents a rationale for tailoring implementation strategies within a values-driven implementation approach. Values-driven implementation seeks to organize implementers around clarifying statements of their shared values in ways that harmonize implementation dynamics related to individual and group mental models, relationships among implementers, and the implementation climate. The proposed approach to tailoring strategies is informed by systems theory and emphasizes the need to focus on both tangible events and behaviors, as well deeper patterns, structures, relationships, and mental models, in order to increase the likelihood of sustaining implementation efforts and improving outcomes for people and communities. We offer for consideration three specific sets of context determinants that are under-represented in the implementation literature and that emerge as especially relevant within a systems approach to identifying and successfully tailoring implementation strategies in the implementation setting including relationships, mental models, and implementation climate.

## Introduction

Change happens in many ways. The science of implementation is essentially a science of change, and as such implementation science focuses on the many and often complicated factors that influence change. An inherent assumption implied by many implementation policies and practices is that implementation is both linear and rational (1, 2). However, implementation in practice is a dynamic and highly relational process involving multiple layers of context and differing norms and values among stakeholders (3).

Given the complexity of implementation, how might we tailor implementation strategies in ways that yield desirable and sustainable outcomes? This paper presents a rationale for tailoring implementation strategies within a values-driven implementation approach. Values-driven implementation seeks to organize implementers around clarifying statements of their shared values in ways that harmonize implementation dynamics related to individual and group mental models, relationships among implementers, and the implementation climate.

Values-driven implementation is informed by the Systems Iceberg Model (4) and Water of Systems Change (5) – two frameworks for leading and supporting transformational systems change. Both of these frameworks diagnose determinants of implementation at three broad levels distinguished as observable events, patterns of interactions that give rise to observable events, and less observable mental models that drive patterns and events over time. The Systems Iceberg Model emphasizes the role of individual and collective mental models as the hidden forces shaping patterns of behavior and beliefs over time. The Water of Systems Change framework demonstrates that systems change requires simultaneous structural, relational, and transformational change, and transformational change requires attention to mental models that drive behavior and belief. When applied to implementation practice these frameworks shine light on actions and behaviors (observables; things we can see); underlying issues (past experiences, relationships, context, needs, and underlying motivations); and foundational and often unexplored mental models that drive behavior and belief (judgements, values, assumptions). The deepest level of both frameworks is where a thorough understanding of the barriers and enablers to implementation is developed. This paper will describe how focusing on *both* tangible events and behaviors, as well deeper patterns, structures, relationships, and mental models, will increase the likelihood of sustaining the systems changes needed to improve outcomes for people and communities. Key terms featured in this paper, and the role these constructs play in using and tailoring implementation strategies, are described in Table 1.

**Table 1:** Key terms.

Term	Definition	Example of Mediating or Moderating Role for Implementation
Values	Qualities deemed important, ideals or standards we hold that govern the decisions we make.	Values of individuals affect the level of buy-in for new interventions (mediator)
		Multi-step, design centered processes can help to identify, define, and iterate on a shared set of values for implementation decision-making.
Principles	Decisions we make based on our values; what it looks like when our values are “in action.”	Principles will determine the actions taken by implementation stakeholders (mediator)
		Principles – values in action – can be assessed during implementation through the following three questions:
		1. How have principles driven decision-making?
		2. How have principles aligned with actions taken?
		3. How have principles informed resource allocation?
Mental Models	A person's mental representation of the way some aspect of the world works, influenced by beliefs and assumptions about the world.	Mental models affect whether implementation stakeholders are motivated to support change efforts (mediator) Researchers can use a systems model can be used to diagnose implementation challenges and determine if challenges are related to mental models:
		1. What are you observing in this challenge?
		2. What may be clouding your vision? What may be clouding the vision of partners?
		3. What may be motivating partners? What is motivating you?
Trusting Relationships	Trusting relationships are centered in vulnerability where the beliefs or expectation of individuals in the relationship are that actions will cause no harm and will provide benefit.	High-quality relationships magnify the impact of implementation strategies on implementation outcomes (moderator)
		Researchers can use measures of psychological safety, trust, and team cohesion to understand the strength of relationships. Qualitative methods are also useful for understanding the quality and impact of relationships on implementation progress.
Implementation Climate	Individual and contextual factors that manifest as openness to change and implementation of new practices	Reduction in resistance to change (moderator)
		Increase in mindsets and behaviors that promote implementaiton of new practices (moderator)

### Systems, systems change, and implementation

Kania, Kramer, and Senge (5) assert that the foundational challenge of systems change is recognizing the systemic forces at play that maintain the current state of the organization or initiative. Specifically, they describe six conditions at three levels that hold organizations and initiatives in place and, therefore, must be addressed to foster systems change. These conditions include policies, practices, and resources (explicit structural change); relationships and power dynamics (semi-explicit relational change), and mental models - a person's mental representation of the way some aspect of the world works - (implicit transformative change).

Unlike the complex inter-relations described by Kania, Kramer, and Senge (5) many research pipeline models assume that the translation of research into practice *via* implementation advances in a stepwise, rational manner (6). These models mask the complexity of the research-practice ecosystems which are characterized by uncertainty, emergence, and embedded unpredictability. Systems change models that embrace complexity (e.g., Systems Iceberg and Water of Systems Change) are poised to support implementation scientists in understanding and describing this complexity. Greenhalgh and colleagues’(7) work on diffusion of innovation integrates many aspects of systems theory including the needs, motivations, values, and social networks of service users, the tension for change and power imbalances within the system, and decision-making authority in the implementation process. However, these variables are not typically considered in selection and tailoring of implementation strategies.

When we ignore the deeper levels of the systems change frameworks we risk implementing change that is not relevant, sustainable, or adequately contextualized for the communities where we work. For example, each level down on the Iceberg – events, patterns of behaviors, systems structures, and mental models - offers a deeper understanding of the system being examined and the strategies for creating change.

Birney (8) describes relational dynamics, values and mental models as the deeper routes to the social tipping points and infrastructure required for sustainable implementation. Implementation methods do not consistently recognize relational and systemic processes, but favor what is tangible, rational, and observable, including individual behavior changes. While individual behavioral changes are a critical aspect of implementation success, transformational change may require paying attention to these other dynamics that might also offer deeper routes to the scale and sustainability of service change needed for population-level improvements and equitable outcomes.

Complex systems consist of dense webs of relationships where individual stakeholders self-organize through interactions. In turn, interactions produce co-learning and collaborative problem solving of complex systems challenges. Several different theoretical models underscore the importance of addressing systemic change, driven by people's values and relationships, when planning for implementation. For example, social capital theory describes how stakeholders access resources from one another through social ties. Indeed, Palinkas and colleagues noted that “successful implementation of evidence-based practices requires consideration and utilization of existing social networks of high-status systems leaders that often cut across service organizations and their geographic jurisdictions” (9).

Additional theories also posit the critical nature of relationships and shared mental models in systems change and implementation efforts. For example, cultural exchange theory describes how the transaction of knowledge among diverse stakeholder groups includes debate, mediation and compromise. Ecological systems theory emphasizes that collaborative efforts of stakeholders are influenced by macro system conditions such as leadership changes and sociopolitical processes. Interactive models of stakeholder involvement in implementation are grounded in experience-based co-design models (10, 11) and co-creation models (12).

Systemic approaches to tailoring implementation strategies invite us “to go underneath the surface of understanding change, to not just look at what is happening above the surface, in the seen behaviors, the tangible events, but to understand the deeper patterns and structural dynamics as well as the mind-sets or worldviews that inform these dynamics and the behaviors we might see” (13). Ignoring these underlying patterns and structures devalues the role they most certainly play in implementation efforts.

### Implementation strategies and tailoring

Much of the science of implementation focuses on frameworks of factors that identify the relations among context, intervention components, implementation strategies, and desired outcomes. Among those factors are implementation strategies that are “the methods or techniques used to enhance the adoption, implementation, and sustainability of a clinical program or practice” (14). The success of any implementation strategy is related to its alignment with causal pathways that lead to desired outcomes (15) and capacity to address known barriers and enablers to implementation (16, 17).

Tailoring implementation strategies involves selecting and modifying implementation strategies based on knowledge of specific barriers and enablers to implementation (18). Selection and tailoring of strategies require complicated methods that often need to be multi-faceted and multi-level in order to address the complexity of services settings and the diverse perspectives of a range of implementation stakeholders (19).

Implementation researchers have identified specific challenges to tailoring strategies that are impediments to achieving implementation outcomes (20, 21). For example, there is a tendency to over-rely on traditional “easy-to-use” and “one-size-fits all” strategies such as training or incentives, or a “kitchen sink” approach where multiple strategies are used without detailed assumptions of how particular strategies will address specific implementation determinants and influence outcomes (22–24). Indeed, several studies demonstrate that there is often a mismatch between the selection of implementation strategies and the implementation barriers these strategies are meant to address. Findings from these studies indicate challenges with identifying appropriate implementation strategies, as well as using these strategies with the appropriate frequency, intensity and fidelity required for these strategies to have their intended benefit (14, 25).

Beyond identified challenges, tailoring of strategies may have limited success because taxonomies of implementation strategies do not sufficiently account for the complex process of implementation. There remains little evidence on how to effectively match implementation strategies to address contextual determinants of implementation such as organizational change and the extent to which leaders are engaged. Waltz and colleagues (21) describe how the identification of barriers and enablers is not sufficient to select and tailor strategies. Selecting and tailoring implementation strategies based on existing taxonomies may not yield sustainable change in complex systems because existing strategies do not address deeper patterns, structural dynamics, and the mindsets or worldviews of stakeholders that inform these dynamics and the behaviors we might see (13). The use of existing strategies, however, is often incentivized by funders and policymakers who seek quick results and do not support timeframes that allow for the use of strategies that may address deeper patterns, relationships, and structural dynamics.

A comprehensive understanding of the implementation problem, inclusive of multiple data sources and perspectives, is needed to effectively tailor strategies to achieve implementation outcomes. Developing a thorough understanding of implementation barriers and enablers is predicated on the ability of implementation scientists to assess contributing factors that are not explicit or readily visible or measurable, including the mental models, values, and assumptions of implementation stakeholders, and the extent to which implementation stakeholders trust each other during the implementation process.

### Tailoring based on A systemic view of implementation determinants

Numerous factors act as determinants of intervention implementation including aspects of the intervention, the setting, the organization, the participants, and the providers (26). It is not surprising therefore that definitions of tailoring implementation have focused on tailoring implementation strategies (19) and tailoring intervention components (18, 27) to address observable determinants of implementation. Nilsen & Bernhardsson (28) reviewed 17 implementation determinant frameworks to describe the scope and nature of context determinants of implementation, and from the review determined that there is meaningful variation in determinants across frameworks as well as a need to identify core elements of implementation contexts that act as barriers and enablers to implementation.

Frequently used implementation frameworks add specificity to the term *context* by identifying dimensions of context that are especially relevant for implementation. For example, the Consolidated Framework for Implementation Research (29) refers to contextual determinants in both the inner and outer implementation setting. Li and colleagues (30) refer to macro, organizational, and local context factors. Macro or outer context refers to socio-political and economic forces that either facilitate or hinder implementation efforts. Inner context includes both organizational and local context and refers to an organization's culture and climate that influence the behavior of individuals and the activities and relationships within the local setting that can also influence implementation (31).

In addition to the determinants identified in the aforementioned established and widely used implementation frameworks, we offer for consideration three specific sets of context determinants that are under-represented in the implementation literature and that emerge as especially relevant within a systems approach to identifying and successfully tailoring implementation strategies in the implementation setting. The three sets of determinants we identify reflect a systemic view of implementation and include as determinants: relationships, mental models, and implementation climate.

### Relationships

Successful implementation includes genuine and meaningful interaction among a range of stakeholders (9, 31–34). For example, focusing on tangible and visible events (first layer of the Iceberg) for stakeholder engagement may result in stakeholders having a “a seat at the table” but not the authentic involvement in implementation decision-making (35) that has been demonstrated to enhance sustainable change (36). Implementation efforts must address the various needs of stakeholders (37). Metz and Bartley (38) found that implementation strategies that promoted relationship-based mutual consultation among stakeholders (second layer of the Iceberg) such as increasing communication, creating a shared understanding of the implementation problem, considering different perspectives, and negotiation led to greater cohesion among stakeholders and increased commitment to the implementation task, consequently leading to improved outcomes.

Metz and colleagues (31, 39) found that high-quality relationships among implementation stakeholders were a critical factor for achieving implementation results. Specifically, the demonstration of trust through various acts – e.g., entering the implementation space with humility as a learner, rather than an expert; engaging in honest and active listening; providing credible information; demonstrating value; demonstrating commitment in the face of complex challenges; staying in difficult situations; showing kindness and vulnerability; demonstrating empathy – was found to positively influence implementation progress. Palinkas and colleagues (40) also describe cultural elements of successful implementation partnerships including flexibility and sensitivity to the needs of individuals in the partnership, openness and honesty associated with building and maintaining trust, and humility and tolerance in service to mutualism and shared understanding of the work.

Relationship-based mutual consultation can elicit information on the different mental models of various implementation actors as it relates to the assumptions and goals of a specific implementation effort. For example, Yazejian and colleagues (41) demonstrated that core competencies for implementation support that supported empathy-driven exchanges, perspective taking, and uncovering mental models of various stakeholders - e.g., co-learning, brokering connections, building trusting relationships - produced added value in the implementation support process and contributed to implementation outcomes such as fidelity and sustainability.

Implementation science has perhaps overly emphasized the selection of strategies to address what we can see – the explicit and observable contextual conditions that determine implementation success – at the expense of recognizing the equal importance of selecting and tailoring strategies specifically to build trusting relationships among implementation stakeholders. For example, implementation strategies that promote sensitivity and responsiveness to the priorities of stakeholders at the implementing site (42) warrant further exploration. Implementation strategies that focus on relationship-based support (addressing the semi-explicit, what is not readily observable) may be more motivational than implementation strategies that address what is observable, such as resources and policies (43). If key stakeholders feel supported, they may feel more hopeful for change even in the face of limited organizational and system resources. This sense of hope may lead to greater commitment and motivation by the stakeholder(s) to pursue change (31).

### Mental models

Implementation efforts often focus on what we can see – tangible events such as training, data collection activities, and meetings – and pay less attention to the “mental models” of leaders, team members, and community stakeholders. Mental models affect the implementation structures put in place, how implementation information is interpreted, how implementation stakeholders interact with each other, and how implementation decisions and investments are made.

Understanding the mental models of various implementation stakeholders can “provide crucial information for understanding, anticipating, and overcoming implementation challenges” (44). Indeed, successful implementation often requires that implementation stakeholders change their mental model - i.e., the values stakeholders hold about the work, the assumptions stakeholders have about how and why outcomes may improve. Therefore, it is critical that strategies are used to elicit mental models, develop awareness of different mental models and assumptions among various stakeholders, and to potentially change mental models.

Mental models can also contribute to the roles people expect to play in implementation. There is evidence that role ambiguity can emerge in early stages of implementation when individuals assert roles that were not previously agreed upon (45). This confusion can actually increase, rather than diminish, as implementation progresses (46). Role ambiguity can limit stakeholders' abilities to improve and sustain the use of evidence-based practices. When stakeholders have a different understanding of their role than those leading implementation, there is evidence that this contributes to communication breakdowns, variability in levels of trust, and some disagreement in decision-making processes or authority (45). Metz and Bartley (38) have identified potential strategies for increasing role clarity among stakeholders, including: frequent feedback loops and communication, the development of a broad understanding of the underlying assumptions for change associated with research evidence, and shared use of data for continuous quality improvement (47).

### Implementation climate

The importance of measuring and influencing organizational climate is well documented in the implementation science literature (48–50), and tailoring implementation strategies to influence or align with organizational culture can contribute to implementation success. However, implementation efforts are often constrained by contextual features such as socio-political-economic conditions (outer context) and issues of power, motivation, and values by stakeholders (local context) indicating that he role of implementation context is salient, multi-level, and in need of specificity.

Nilsen & Bernhardsson (28) identified two broad categories of context determinants; those aspects of the context that are absolute determinants (e.g., funding and resources) and those that are influential drivers of successful implementation (e.g., shared agendas and relationships).

Implementation scientists focused on contextual factors related to shared agendas and relationships use the term *implementation climate* (51) to describe the contextual forces that shape implementers' beliefs about the value and support for an intervention. Helfrich and colleagues (52) specified that implementation climate is comprised of context factors that manifest as collective value for and receptivity to the intervention and recommend the use of standardized tools to assess implementation climate in an effort to provide tailored supports. Tailoring for implementation climate requires a deeper focus on measurable, as well as challenging to measure, factors that can serve as strong barriers or enablers of sustainable change such as implementers' beliefs about the individual and institutional value and support for the intervention to be implemented (51, 53, 54).

### Intersectionality of relationships, mental models, and implementation climate

Tailoring implementation strategies requires a deep understanding of the intersectionality of these three determinants – relationships, mental models, and implementation climate. For example, we know that in many instances, collaborations among stakeholders, including researchers and community members, are strained by a lack of mutual understanding of each other's goals (55). Expectations relate directly to the importance of mental models and identifying determinants such as motivation and values that may serve as barriers and enablers to implementation progress. Therefore, tailoring implementation strategies for contextual determinants (e.g., organizational climate) should also include relationships among stakeholder groups as well as mental models that exist within individuals and aggregate upwards to group dynamics based on shared and often unexplored mental models of how and under what conditions change happens.

### Values-Driven implementation

**Figure 1:**
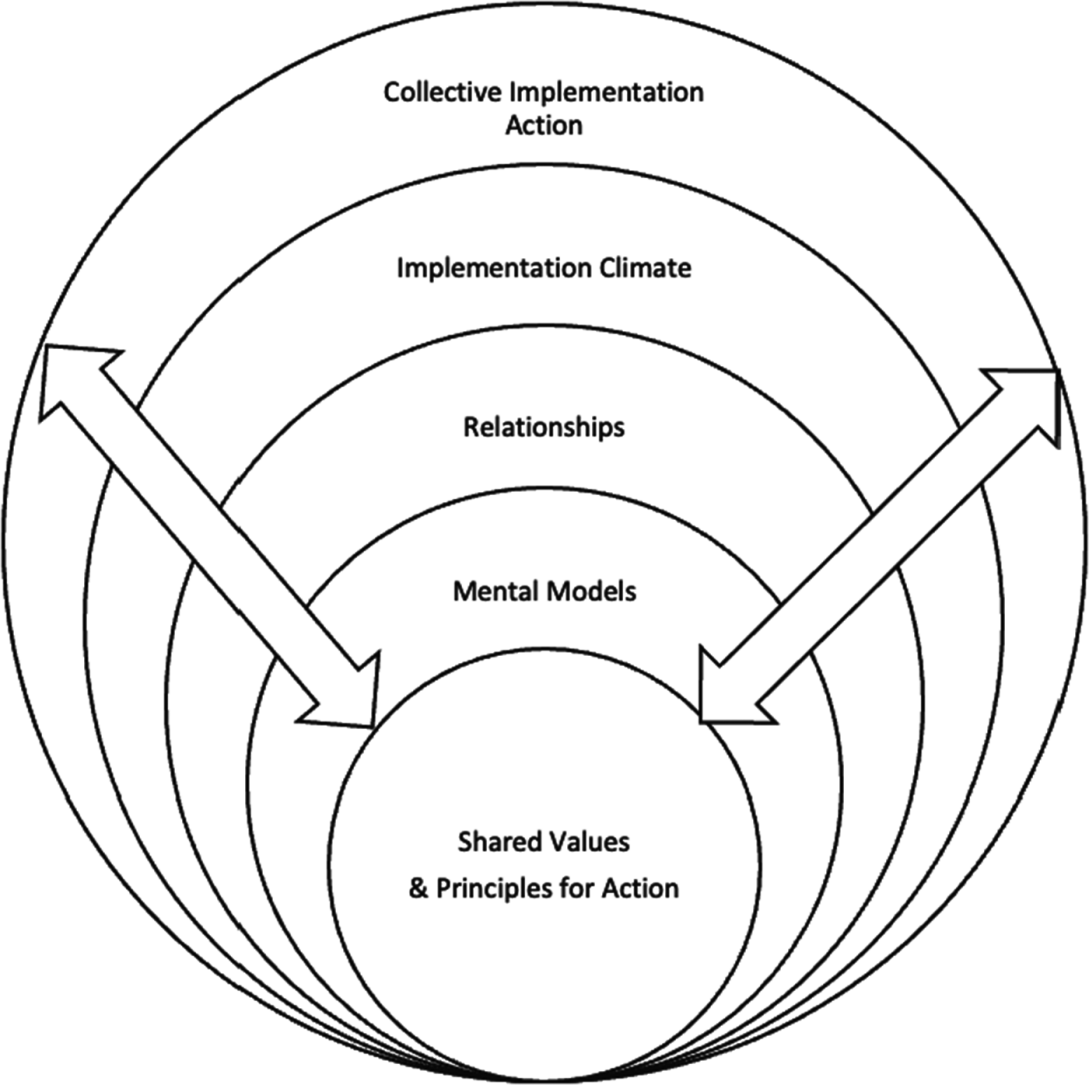
Shared values anchor and harmonize the complex and multi-level interaction of mental models, relationships, and implementation climate as they build toward collective implementation action.

Considering the convergent evidence indicating that implementation climate (52) and context, including drivers of implementation (28), are powerful determinants of implementation we believe that methods are needed to promote conducive contexts and collective behavior during the implementation process. Standardized tools to assess implementation climate will be beneficial, but more specific methods to promote positive implementation climate are needed as well. Such methods will work with the individual and collective mental models that form people's beliefs about change as well as the conditions that affect implementation climate and the quality of relationships among implementers and participants (Figure 1).

Individual and collective mental models interact with aspects of implementation climate and inter-relationships to form the conditions for collective implementation action. Toward that end we provide examples of work using organizational values and principles as tools for tailoring to harmonize mental models, relationships, and implementation climate. Generally speaking, *values* refer to qualities deemed important, ideals, or standards we hold that govern the decisions we make (56). The term *principles* refer to decisions about what to do based on our values – what it looks like when our values are “in action.” Principles guide choices about desirable behavior. Principles can be a rudder for navigating complex adaptive systems and, when understood and applied to organizational decision-making, can guide us toward our desired results (56). Values operate multi-dimensionally, existing at the individual level and aggregating up to the organizational level (57). Once identified, understood, and shared, organizational values can drive the creation of principles suitable for guiding organizational decision-making and action in multiple areas of systems change.

Values and principles are often not explicitly identified or used to guide systems change efforts from the outset of implementation. In fact, organizational or community values are often described as barriers to implementation, rather than as guideposts to implementation, in the implementation science literature (21, 58). For example, implementation research is often concerned with stimulating changes to contextual variables, including the values and attitudes of direct practitioners, to align with the use of evidence-based programs (59), rather than using values of practitioners and community members to drive the selection of intervention and services in communities. We recommend the use of values and principles to inform the selection and tailoring of implementation strategies, decision-making, and course corrections in the implementation setting. Values-focused implementation seeks to unearth, engage, and connect people's mental models so that through identification and application of shared values, groups might arrive at the changes in relationships, power dynamics, resources, policies, and practices needed to foster meaningful and equitable improvements.

Selecting implementation strategies based on principles has many benefits when working in complex systems:

• Principles inform implementation choices.
• Principles are grounded in values and what matters to those who have the greatest stake in implementation.
• Principles provide direction on implementation strategies and allow tailoring and adaptation of implementation strategies along the way.
• Principles can be applied contextually to ensure relevance.
• Principles can serve as a guide for implementation in complex and complicated systems (when we are not implementing a single EBP within an organization but are seeking systems change through multiple workstreams).

Successful implementation is the product of numerous shared decisions. In implementation efforts, opportunities exist for critical decision-making that can either increase or decrease the likelihood that implementation will result in desired outcomes. Relying on values and principles, which are defined by the community, propels implementation leaders and teams to engage in deliberate and transparent decision-making. Implementation decisions should be conscious, reflective, well-thought-through, and paced in a way that unintended consequences can be assessed. By vetting all decisions and actions through the lens of values and principles, we can increase the likelihood that decision-making is transparently communicated with stakeholders at all levels of implementation.

In addition, values and principles can form an anchor for decision-making that increases the fitness of groups as they respond to challenges and changes in the dynamic implementation space. At the outset of an implementation initiative shared values serve as a readiness component for change initiatives (60). Further, in complex, place-based interventions, learning occurs during initial implementation, and that learning can be used to generate improved implementation processes (communications, networks, routines, supports) to achieve desired practice and outcomes (61). Learning informed by community values help adaptive implementation responses to reinforce, rather than disintegrate, momentum toward desired outcomes. This is accomplished by using principles to guide decisions and subsequent implementation action.

Successful implementation of anything – a program, practice, policy, or strategy – requires an infrastructure that supports the development of staff skills and sustainable practice, organizational, and systems change. The use of values and principles to guide implementation efforts is no exception. Leaders and teams supporting implementation efforts will need to develop their own competency to use values and principles in implementation and improvement activities. For example, team leaders and champions will need to develop knowledge of the values and principles, as well as skills to support others in using the values and principles in everyday implementation activities (e.g., facilitation and reflective practice skills).

Organizational and systems supports that create a hospitable environment for the values and principles to be used are also needed. For example, funding and evaluation resources may be needed to enable teams to gather data, test hypotheses, and conduct research that will increase the likelihood that their decision-making is aligned to values and principles. Teams may also need additional resources to develop and implement communication protocols and feedback loops with community members and other key stakeholders to increase the alignment of their work with the community's values and principles.

**Figure 2:**
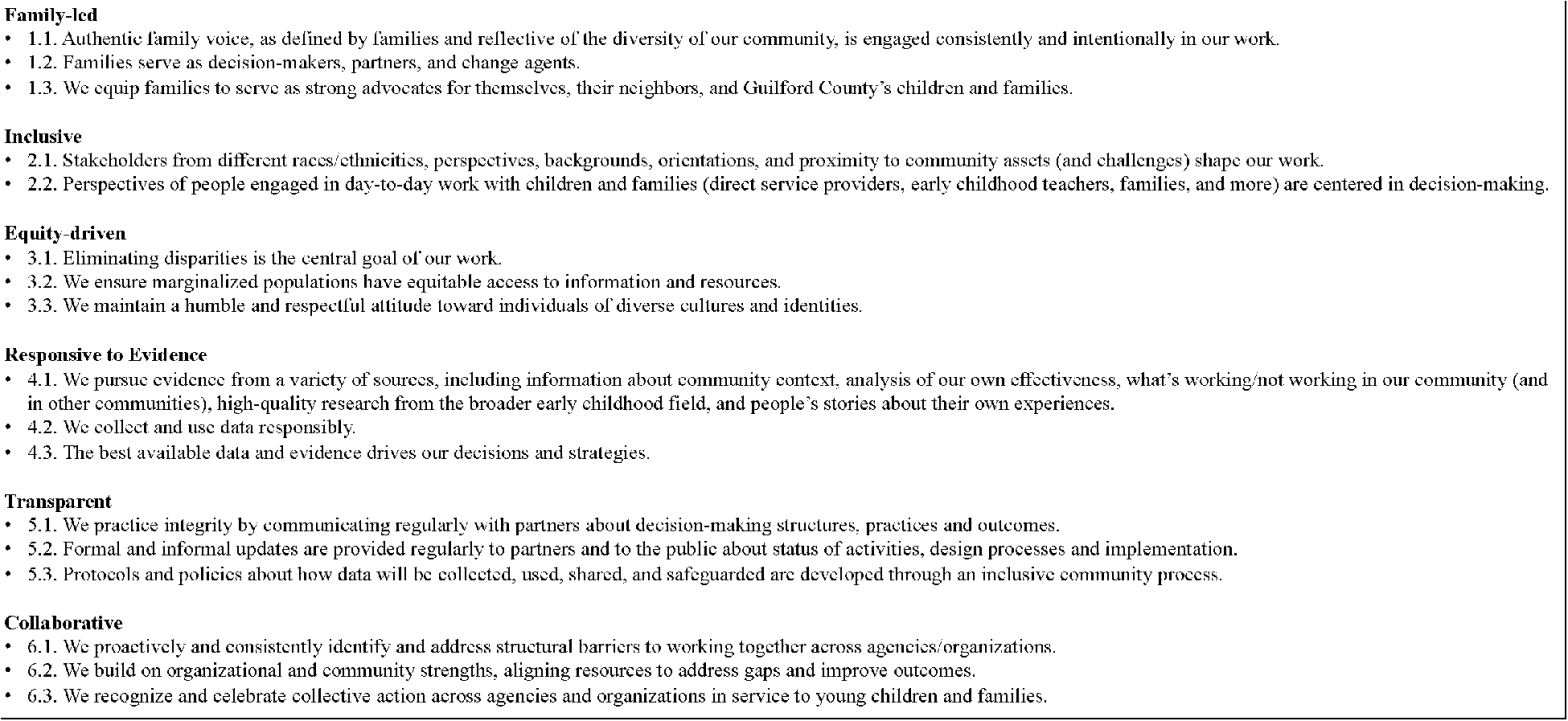
Example of Values and Principles Guiding Implementation of Initiative to Improve Outcomes for Children Ages 0–8.

In our experience supporting implementation efforts we have observed the harmonizing effect of group values and principles in application. We provide an example of one agency's values and principles in Figure 2. Community stakeholders developed and vetted the goals and principles over the course of six months. Achieving shared values included: 1) facilitating design-centered activities centering perspectives of those individuals with lived experience and using collective sense-making and negotiation; 2) supporting co-development of values and principles; 3) working with community members to build a strong fit between values (beliefs) and principles (actions based on beliefs) and goals of the implementation effort; and 4) iteratively improving initial prototypes of values and principles by testing their application in implementation decision-making and resource allocation. Once developed program staff and primary partners with implementation roles used the values and principles as rudders for action and decision-making during the implementation process. As implementation progressed questions about the legitimacy and relevance of implementation activities were scrutinized through the lens of the values and principles, allowing for decisions to be framed, revised, and communicated in alignment with the organization's stated values.

Implementation teams may want to start with defining values first, seeking consensus on the values before taking a deeper dive into the principles. Because principles refer to the decisions and actions we take based on our beliefs (or values), groups and organizations may find it easier to reach agreement on values before taking the time to define the principles that align with each value. If this process is successful, it is more likely that team members and implementation stakeholders will report that they feel heard, they had a say in crafting the values, they are a valued partner in the work, and they invested their time well. These processes are important for internal implementation team members at an organization, as well as with implementation stakeholders in community change efforts. Many implementation efforts in organizations will have impacts on stakeholders outside of the organization. In these cases, processes to develop shared values and principles should involve internal team members and community partners.

### Selecting and tailoring strategies for values-driven implementation

Values-driven implementation requires the selection and tailoring of strategies to elicit and understand values of implementation stakeholders, to build relationships aligned with these values, and to support an implementation climate that facilitates the use of these values in decision-making and ongoing improvement of implementation efforts. We propose that tailoring implementation strategies based on values requires tailoring in three areas: 1) understanding individual and group mental models and choosing strategies that influence such mental models to strengthen alignment with values; 2) tailoring strategies to build relationships that represent shared values; and 3) tailoring strategies to foster an implementation climate that promotes values-driven implementation. Below we provide examples for reflecting, understanding and tailoring strategies within each of these areas.

### Selecting and tailoring strategies for individual and group mental models

Effective implementation support requires understanding the values, beliefs and assumptions of individuals and group members who have a stake in the implementation effort. Implementation efforts often involve implementation teams - a group of stakeholders, including community members, that oversees, attends to, and is accountable for, performing key functions in selecting, tailoring, and improving implementation strategies (62). In order for implementation teams to be effective the group must develop relational cohesion – the perception by individuals on a team that their relationships are a unifying element or force in the implementation effort (63, 64).

Relational cohesion can be developed through tailoring strategies that elicit an understanding of individual team members’ values and assumptions about the implementation effort. For example, supporting co-learning is an example of how tailoring implementation strategies can be used to understand and change individual and group mental models. Specifically, implementation strategies can be used to support implementation stakeholders to communicate and listen for the purpose of mutual understanding and the collaborative integration of different perspectives and types of knowledge.

Successful implementation is a collaborative act relying on multiple actors in iterative and unpredictable ways (65). In order for implementation to be successful and sustainable, new ways of thinking are required to select and tailor strategies that specifically promote collective action. Eliciting, understanding, and influencing mental models is a critical first step in the tailoring process.

### Selecting and tailoring strategies for relationship qualities

Implementation literature describes relationships as foundational for effective implementation (38, 46, 66–69); however, tailoring implementation strategies to build relationships among stakeholder groups is not typically discussed. Metz and colleagues (31) have reported a range of strategies that are used to build relationships including engagement, facilitation, collaboration, and consensus building. Trust has also been identified as a critical relationship quality for implementation success (31, 34, 70). There are several implementation strategies that have described as both a contributor to and an outcome of trust including collaboration (71), communication (38), empathy (39), and sensitivity (42).

Metz and colleagues (34) describe relational implementation strategies as strategies undertaken to build trust through strengthening the quality, mutuality, and reciprocity of interactions among implementation stakeholders. High quality relationships can magnify the impact of other implementation strategies as part of the tailoring process. Developing a comprehensive understanding of implementation barriers and enablers is an important step in the tailoring process, specifically for building high quality relationships. Correctly diagnosing implementation challenges can allow for the appropriate selection of strategies that can be used to build trusting relationships, thereby moderating the impact of other strategies on the implementation problem.

### Selecting and tailoring strategies for implementation climate

Implementation scientists have identified key methods for tailoring implementation strategies to address variation in implementation contexts (21, 72, 73). Powell and colleagues (20) identified four promising methods from the literature: concept mapping, group model building, conjoint analysis, and intervention mapping. The four methods for tailoring implementation strategies to fit context serve to foster collective understanding of and motivation for implementing an evidence-based practice.

Fostering understanding and motivation are important efforts to build a conducive implementation climate, where climate is defined as the degree to which implementers perceive that a change is expected, supported, and rewarded within the context (51, 53). Perceptions of expectedness, support, and reward vary across individuals in implementation settings (54), and as such the act of tailoring strategies to foster implementation climate requires attention to individual and group dynamics.

The emphasis on tailoring for implementation climate acknowledges that no intervention nor implementation strategy is universally effective. Rather, effectiveness is the multiplicative product of the fit among intervention components and factors in the context and its members that form the determinants of implementation. Values driven implementation harmonizes individual and group sense-making around the identification of and reflection on shared values. Effective values-driven implementation will incorporate participants' ideas about expectedness, support, and reward, ensuring that the selection and application of implementation strategies is consistent with shared values and that implementing according to shared values is expected, supported, and rewarded in the implementation setting.

## Discussion

Selecting and tailoring implementation strategies that are relevant to a particular context and change effort has been described as a major challenge in high quality and consistent implementation of evidence-informed programs, practices and policies (20). These challenges have been attributed to a lack of empirical literature on tailoring methods, the underutilization of conceptual and theoretical models for selecting and tailoring implementation strategies, and the variations in service settings and communities where implementation takes place. At the same time, there is a growing call for recognizing the role that systems theory can play in selecting, tailoring, and continuously improving implementation strategies.

We take the position that specific frameworks based on systems theory offer new and important considerations for implementation practice, especially for tailoring implementation strategies. These frameworks – Systems Iceberg Model and Water for Systems Change – embrace the complexity, uncertainty, and emergent nature of most implementation settings, and emphasize the need to select and tailor strategies that address relational dynamics, values, and mental models of implementation actors – the contextual variables that often go unnoticed in implementation planning and research. Chief among these difficult to observe factors of context are individual and organizational values.

This paper presents a rationale for tailoring implementation strategies within a values-driven implementation approach that emphasizes tailoring across three salient and often overlooked dimensions of implementation – relationships, mental models, and implementation climate. In combination these three constructs represent multi-level dimensions of implementation that affect and are affected by values held by individuals and groups.

Trusting relationships have been demonstrated as foundational for implementation efforts, and several strategies have already been identified as promoting trust including frequent interactions, responsiveness, empathy-driven exchanges, and co-learning (31, 34, 70). Selecting and tailoring strategies to foster trust among a range of implementation stakeholder will most likely yield increased commitment and resilience for implementation efforts.

Mental models represent the values, beliefs, and assumptions implementation stakeholders hold about the implementation effort. Systems change models emphasize the critical role mental models play in the decision-making and investments that affect the uptake and sustainability of change efforts. Developing a comprehensive understanding of the range of mental models influencing stakeholder interactions is a first step in selecting and tailoring implementation strategies that can seek to develop a shared mental model – or group mental model – for implementation efforts. Mental models are often not accounted for when assessing implementation barriers and enablers, and, consequently, implementation strategies are not tailored to bridge the gap among different mental models for various implementation stakeholders.

Beyond individual mental models, we recognize the shaping functions of shared stories that can serve as silent drivers of group behavior. Our focus on exploring and explicating values is more specifically a focus on learning about the stories that people tell themselves and each other that drive their preferences for interventions, implementation practices, and outcomes. Shared stories allow for intervention and implementation preferences to remain un- or under-explored in the intervention setting because the stories serve as explanatory short-cuts that assuage the *whys* and *hows* of implementation. In a context of un-explored stories, variation in mental models – which can be inevitable and not at all undesirable – can challenge effective implementation. We propose that exploring and expicating values can serve to harmonize implementation factors without requiring that all members of the implementation setting share identical mental models of intervention, implementation, and outcome.

Finally, as we tailor implementation strategies to address barriers or align with synergistic aspects of the implementation climate, it is important to recognize that the implementation climate represents a culmination of the socio-political-economic conditions that are exerting influence on the implementation setting. Explicitly tailoring strategies for the implementation climate can increase the likelihood that implementation strategies will address critical issue of power, motivation, and values among key stakeholders. We also recognize that endeavoring to engage in authentic co-creation that creates reciprocal dialogue among all stakeholders (74) as part of the implementation process is challenging and complex, and most likely a contributing factor as to why implementation strategies are often not tailored to the values of those with the greatest stake in implementation efforts.

Many implementation frameworks, such as the Consolidated Framework for Implementation Research (29), have described how contextual factors such as culture and climate, readiness, and relationships are interrelated and influence implementation efforts. Implementation theories such as the Theoretical Domains Framework (75) have explored how cognitive, affective, social and environmental influences on behavior changes are associated with implementation efforts. We suggest building on the observations, insights, and hypotheses of earlier implementation theories, models, and frameworks to extend and to make more explicit the role values can play in tailoring implementation strategies.

We propose that meaningful advances in implementation legitimacy, impact, and sustainability will be achieved by tailoring implementation strategies with a values-driven approach that explicitly investigates and harmonizes key relationships, active mental models, and implementation climate. Tailoring strategies through values-driven implementation will foster coherence in clarifying implementation goals, developing implementation plans, measuring implementation benchmarks and progress, continuously improving implementation strategies, and assessing implementation outcomes. The work of implementation is inherently complicated and messy. Defining a shared set of values at the outset, with those most affected by the implementation effort, provides a compass for decision-making and a set of guideposts for building relationships, developing a shared mental model for the work ahead, and understanding and addressing barriers related to the implementation climate.

## Data Availability

The original contributions presented in the study are included in the article/Supplementary Material, further inquiries can be directed to the corresponding author.
